# Editorial: Biopsychosocial complexity research

**DOI:** 10.3389/fpsyt.2023.1157217

**Published:** 2023-03-03

**Authors:** Christian Schubert, William Sulis, Alejandro De La Torre-Luque, Günter K. Schiepek

**Affiliations:** ^1^Department of Psychiatry, Psychotherapy, Psychosomatics and Medical Psychology, Medical University of Innsbruck, Innsbruck, Austria; ^2^Department of Psychiatry and Behavioural Neurosciences, McMaster University, Hamilton, ON, Canada; ^3^Department of Legal Medicine, Psychiatry and Pathology, Complutense University of Madrid, Madrid, Spain; ^4^University Hospital of Psychiatry, Psychotherapy and Psychosomatics, Paracelsus Medical University, Salzburg, Austria

**Keywords:** biopsychosocial (BPS) model, systems theory, biosemiotics, complex adaptive systems, psychoneuroimmunology

Advancements in research over the past century have clearly helped people suffering from psychiatric disorders. However, many of these advancements have been based on a mechanistic-reductionistic approach consisting of traditional research designs and methods (e.g., randomized controlled trials, standardized questionnaires) that often fail to capture the full extent of human complexity. This may at least in part explain the inconsistencies, e.g., in conventional stress research, and the poor generalizability from laboratory to real life, naturalistic contexts (1, 2).

Emerging evidence suggests that a paradigmatic shift to the biopsychosocial model of life (3) employing a biosemiotic-systemic approach (4) may accelerate progress in areas where a mechanistic-reductionistic approach has not been successful. To this end, general systems theory (5) can be used as a framework for biosemiotic-systemic thinking. It proposes that humans are deeply embedded in their environment and affected by the continuous influx of stimuli provided by nature, nurture and culture. Moreover, this human-environment entity is hierarchically structured, consisting of various vertically stratified levels of systems such as molecular, cellular, tissues, organs, person, relationship, family, population and ecosystem (6) (Figure 1).

**Figure 1:**
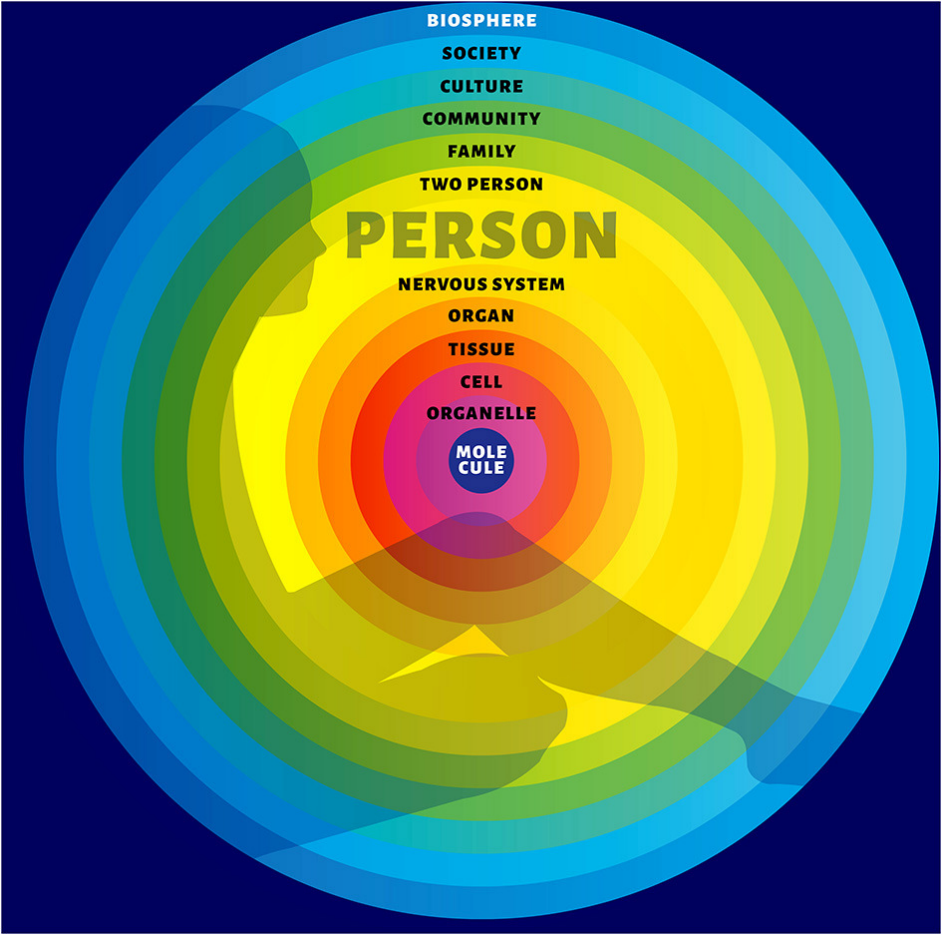
Biopsychosocial human-environment entity.

From a systemic standpoint, dynamic complexity research suggests that environmental adaptation is connected with order transitions from lower to higher complexity levels. This transition is associated with new distinctive qualities and relationships, e.g., the psyche emerges from neuronal activity. In turn, these higher levels are superordinate to lower levels and set the boundary conditions for them (3, 5, 7). Such intersystemic activity features top-down/bottom-up regulatory circuits that are flexible enough to maintain the hierarchy's systemic integrity in the presence of stress. However, when stress is too great, this equilibrium is disturbed, leading to dysfunctional and disordered activity affecting all levels simultaneously in a contextually dependent manner (8).

In addition, from a biosemiotic standpoint, life evolves through continuous production, exchange and interpretation of signs at all levels of the biopsychosocial hierarchy (9). On the person level, the interpretation of a sign, e.g., a social stressor, is always connected to the whole biography of the person, including conscious and unconscious as well as objective and subjective factors. According to biosemiotics it is the subjective meaning a sign has for the interpreting person that determines how the person as a whole responds to that sign and whether the process of interpretation, i.e., the assignment of meaning, is good or bad for health (4, 9).

From these introductory remarks, it should be clear that simply paying attention to diversity in biopsychosocial research and merely combining biological, psychological and social data sets cannot do justice to the complexity of human existence (10). But how can research be conducted such that it is able to match the complexities of the biopsychosocial model of life? We asked this question about two years ago as part of a Frontiers Research Topic and received five thematically relevant papers, of which four are theoretical and one empirical.

Sturmberg's paper introduces the field of complex pattern formation in disease and how it can be used to improve patient management. It then offers various perspectives supporting the philosophical/theoretical proposition of the complex-adaptive nature of health, i.e., Ashby's law of requisite variety, multiple sufficient causes, network physiology, inflammatory regulation, top-down causation in complex adaptive systems. The article also presents an outlook on how a new paradigmatic view of dynamic complex-adaptive states could alter health system practice and research to become more suitable with regard to the person as a whole.

Two papers in this Research Topic (Sulis and Trofimova) propose a continuum from temperament to mental illness and formal ways to analyze it. Both papers argue that a mathematics (or physics) based upon timeless, fixed structures and symmetries cannot express the complexity of organisms in which transience, emergence, generativity and contextuality abound. Sulis views the continuum as a landscape of transient dynamical phases, generalizing ideas of dynamic systems theory through the concept of a generating process.

Trofimova approaches the continuum from the perspective of functional constructivism, outlining universal features in the construction of behavior, from which is derived the neurochemical framework Functional Ensemble of Temperament (FET). A spectral approach to classification of temperament traits and symptoms of psychopathology is presented in the FET, based on neurochemical biomarkers. Moreover, Trofimova suggests using the concept of Specialized Extended Phenotype (SEP) to highlight the mechanisms of multi-level reinforcement of psychobehavioral diversity. Biopsychosocial complexity, therefore, could be partitioned as types of context and SEP functional “bubbles” using the same 12 categories as 12 neurochemical FET components.

The paper by Lunansky et al. follows a network approach to psychiatry where psychopathology emerges from causally interacting symptoms. It presents three studies (two simulation studies, one with empirical data) dealing with a formal system of interacting psychiatric symptoms targeted by biopsychosocial risk and protective factors to influence resilience. The studies applied two novel network resilience metrics, the Expected Symptom Activity (ESA), indicating how many symptoms are active or inactive, and the Symptom Activity Stability (SAS), indicating how stable the symptom activity patterns are.

Finally, the paper that used an empirical approach to biopsychosocial complexity is from Seizer et al. and re-evaluates an “integrative single-case study.” In this study on a 25-year-old healthy woman, a dynamic complexity measure was applied to biopsychosocial time series data covering a study period of 126 12-h intervals under “life as is it lived” conditions. It was shown that the about-weekly pattern in the subject's cellular immune complexity (indicated by neopterin) was an expression of a whole-person adaptation toward the emotionally meaningful in-depth interviews during the 2-month period. This study supports the notion that integrating time and meaning in research methodology gives access to the full richness of a person's complex biopsychosocial reality.

Taken together, the contributions of this Research Topic show that considering complexity in biopsychosocial research should allow psychiatry to explore new horizons. This, however, will require a fundamental epistemological shift toward a biosemiotic-systemic paradigm in medicine (1–3).

## Author contributions

CS and WS wrote the manuscript. All authors provided important intellectual contributions, read, and approved the final version.
